# Supplementary material for: Muscle architecture, voluntary activation, and low-frequency fatigue do not explain the greater fatigue of older compared with young women during high-velocity contractions
Source: PLoS One. 2020 Nov 3;15(11):e0234217. doi: 10.1371/journal.pone.0234217 (PMC7608879; doi:10.1371/journal.pone.0234217)
Supplement: S1 Table — Values are mean±SE. 95% CI, 95% confidence intervals for the difference between group means; 0R, zero recovery (immediately following the fatiguing contraction protocol); 5R, 5-min recovery; 10R, 10min-recovery; 20R, 20-min recovery; 30R, 30-min recovery. (DOCX) [file pone.0234217.s001.docx]

| **S1 Table. Age-related differences in the peak rate of torque development** | | | | | |
| --- | --- | --- | --- | --- | --- |
|  | Young (n=8) | Older (n=8) | 95% CI | *P* | *Effect size* |
| Rate of torque development (%pk**^.^**ms^-1^) | | | | | |
| 0R | 1.29 ± 0.09 | 1.08 ± 0.07 | -0.46, 0.04 | 0.090 | 0.89 |
| 5R | 1.31 ± 0.10 | 1.20 ± 0.07 | -0.36, 0.14 | 0.353 | 0.47 |
| 10R | 1.35 ± 0.09 | 1.23 ± 0.11 | -0.44, 0.19 | 0.407 | 0.41 |
| 20R | 1.30 ± 0.11 | 1.24 ± 0.16 | -0.48, 0.36 | 0.7549 | 0.15 |
| 30R | 1.34 ± 0.12 | 1.24 ± 0.14 | -0.51, 0.31 | 0.605 | 0.26 |
| Values are mean±SE. 95% CI, 95 % confidence intervals for the difference between group means; 0R, zero recovery (immediately following the fatiguing contraction protocol); 5R, 5-min recovery; 10R, 10min-recovery; 20R, 20-min recovery; 30R, 30-min recovery. | | | | | |
