# Supplementary material for: Muscle architecture, voluntary activation, and low-frequency fatigue do not explain the greater fatigue of older compared with young women during high-velocity contractions
Source: PLoS One. 2020 Nov 3;15(11):e0234217. doi: 10.1371/journal.pone.0234217 (PMC7608879; doi:10.1371/journal.pone.0234217)
Supplement: Attachment — Submitted filename: Response to Reviewers.docx [file pone.0234217.s002.docx]

Dear Dr. Mirkov,

We thank you and the Reviewers for taking the time to read our manuscript and provide insightful and critical feedback. Our responses to each of the Reviewer’s comments are shown in rent font below each individual comment.

Best regards,

Dr. Liam F. Fitzgerald

Dear Dr. Fitzgerald,

Thank you for submitting your manuscript to PLOS ONE. After careful consideration, we feel that it has merit but does not fully meet PLOS ONE’s publication criteria as it currently stands. Therefore, we invite you to submit a revised version of the manuscript that addresses the points raised during the review process.


Please submit your revised manuscript by Oct 09 2020 11:59PM. If you will need more time than this to complete your revisions, please reply to this message or contact the journal office at [plosone@plos.org](mailto:plosone@plos.org). When you're ready to submit your revision, log on to [https://www.editorialmanager.com/pone/](https://urldefense.proofpoint.com/v2/url?u=https-3A__www.editorialmanager.com_pone_&d=DwQGaQ&c=sJ6xIWYx-zLMB3EPkvcnVg&r=khMOZAIPugRDx2mPk882SyrBxYwUZMrH0Ns3Ow2XUos&m=UeqZnOL9tqkQWNIWl4V2FUZEUT3QfURydfRinmczEi8&s=E91yF83Gh2nblboLklAm0sCKO-fabsLzdea-cceDs6U&e=) and select the 'Submissions Needing Revision' folder to locate your manuscript file.

Please include the following items when submitting your revised manuscript:

- A rebuttal letter that responds to each point raised by the academic editor and reviewer(s). You should upload this letter as a separate file labeled 'Response to Reviewers'.
- A marked-up copy of your manuscript that highlights changes made to the original version. You should upload this as a separate file labeled 'Revised Manuscript with Track Changes'.
- An unmarked version of your revised paper without tracked changes. You should upload this as a separate file labeled 'Manuscript'.

If you would like to make changes to your financial disclosure, please include your updated statement in your cover letter. Guidelines for resubmitting your figure files are available below the reviewer comments at the end of this letter.

If applicable, we recommend that you deposit your laboratory protocols in protocols.io to enhance the reproducibility of your results. Protocols.io assigns your protocol its own identifier (DOI) so that it can be cited independently in the future. For instructions see: [http://journals.plos.org/plosone/s/submission-guidelines#loc-laboratory-protocols](https://urldefense.proofpoint.com/v2/url?u=http-3A__journals.plos.org_plosone_s_submission-2Dguidelines-23loc-2Dlaboratory-2Dprotocols&d=DwMGaQ&c=sJ6xIWYx-zLMB3EPkvcnVg&r=khMOZAIPugRDx2mPk882SyrBxYwUZMrH0Ns3Ow2XUos&m=UeqZnOL9tqkQWNIWl4V2FUZEUT3QfURydfRinmczEi8&s=bmf2nTb3Pynp3NiTgWGSzljw661E7KUKMJe8yOmbOL8&e=)

We look forward to receiving your revised manuscript.

Kind regards,

Dragan Mirkov, Ph.D.
Academic Editor
PLOS ONE

Journal Requirements:

When submitting your revision, we need you to address these additional requirements.

1. Please ensure that your manuscript meets PLOS ONE's style requirements, including those for file naming. The PLOS ONE style templates can be found at

[https://journals.plos.org/plosone/s/file?id=wjVg/PLOSOne_formatting_sample_main_body.pdf](https://urldefense.proofpoint.com/v2/url?u=https-3A__journals.plos.org_plosone_s_file-3Fid-3DwjVg_PLOSOne-5Fformatting-5Fsample-5Fmain-5Fbody.pdf&d=DwQGaQ&c=sJ6xIWYx-zLMB3EPkvcnVg&r=khMOZAIPugRDx2mPk882SyrBxYwUZMrH0Ns3Ow2XUos&m=UeqZnOL9tqkQWNIWl4V2FUZEUT3QfURydfRinmczEi8&s=LotFYHF_i-wadmhmx5BsVldjkU6JYbrK97sDUaVXWHU&e=) and

[https://journals.plos.org/plosone/s/file?id=ba62/PLOSOne_formatting_sample_title_authors_affiliations.pdf](https://urldefense.proofpoint.com/v2/url?u=https-3A__journals.plos.org_plosone_s_file-3Fid-3Dba62_PLOSOne-5Fformatting-5Fsample-5Ftitle-5Fauthors-5Faffiliations.pdf&d=DwQGaQ&c=sJ6xIWYx-zLMB3EPkvcnVg&r=khMOZAIPugRDx2mPk882SyrBxYwUZMrH0Ns3Ow2XUos&m=UeqZnOL9tqkQWNIWl4V2FUZEUT3QfURydfRinmczEi8&s=6KLwLRq5_HR8CHRAwNkGF7Oosxjgp487svbz2H5XLe8&e=)

2. We note that you have included the phrase “data not shown” in your manuscript. Unfortunately, this does not meet our data sharing requirements. PLOS does not permit references to inaccessible data. We require that authors provide all relevant data within the paper, Supporting Information files, or in an acceptable, public repository. Please add a citation to support this phrase or upload the data that corresponds with these findings to a stable repository (such as Figshare or Dryad) and provide and URLs, DOIs, or accession numbers that may be used to access these data. Or, if the data are not a core part of the research being presented in your study, we ask that you remove the phrase that refers to these data.

[Note: HTML markup is below. Please do not edit.]

Reviewers' comments:

Reviewer's Responses to Questions

**Comments to the Author**

1. Is the manuscript technically sound, and do the data support the conclusions?

The manuscript must describe a technically sound piece of scientific research with data that supports the conclusions. Experiments must have been conducted rigorously, with appropriate controls, replication, and sample sizes. The conclusions must be drawn appropriately based on the data presented.

Reviewer #1: Yes

Reviewer #2: Partly

2. Has the statistical analysis been performed appropriately and rigorously?

Reviewer #1: Yes

Reviewer #2: Yes

3. Have the authors made all data underlying the findings in their manuscript fully available?

The [PLOS Data policy](https://urldefense.proofpoint.com/v2/url?u=http-3A__www.plosone.org_static_policies.action-23sharing&d=DwMGaQ&c=sJ6xIWYx-zLMB3EPkvcnVg&r=khMOZAIPugRDx2mPk882SyrBxYwUZMrH0Ns3Ow2XUos&m=UeqZnOL9tqkQWNIWl4V2FUZEUT3QfURydfRinmczEi8&s=Jq5aqOwLLPXH7itx68g4xULu-zqdtwqvrWT_nyUaoe4&e=) requires authors to make all data underlying the findings described in their manuscript fully available without restriction, with rare exception (please refer to the Data Availability Statement in the manuscript PDF file). The data should be provided as part of the manuscript or its supporting information, or deposited to a public repository. For example, in addition to summary statistics, the data points behind means, medians and variance measures should be available. If there are restrictions on publicly sharing data—e.g. participant privacy or use of data from a third party—those must be specified.

Reviewer #1: Yes

Reviewer #2: Yes

4. Is the manuscript presented in an intelligible fashion and written in standard English?

PLOS ONE does not copyedit accepted manuscripts, so the language in submitted articles must be clear, correct, and unambiguous. Any typographical or grammatical errors should be corrected at revision, so please note any specific errors here.

Reviewer #1: Yes

Reviewer #2: Yes

5. Review Comments to the Author

Please use the space provided to explain your answers to the questions above. You may also include additional comments for the author, including concerns about dual publication, research ethics, or publication ethics. (Please upload your review as an attachment if it exceeds 20,000 characters)

Reviewer #1: Overall, this is a clear, concise, and well-written manuscript. The introduction is relevant and theory based. Sufficient information about the previous study findings is presented for readers to follow the present study rationale and procedures. The methods are generally appropriate. In essence, the methods part is too long. I suggest simplifying it. Also, this applies to results part. However, results are well presented. Discussion is appropriate, mainly regarding the implications of the findings of the study.

Thank you for your enthusiasm in our manuscript. Based on your feedback, we have revised the methods section to be more concise.

Reviewer #2: The aim of this research paper was to examine the potential roles of resting muscle architecture and baseline contractile properties, as well as changes in voluntary activation and low-frequency fatigue in response to high-velocity knee extensor work. While the authors purposefully included only female participants to avoid gender-related interactions, I believe it would be of interest to compare them with males particularly since the previous results are ambiguous. Also, while it is good that you used G power to determine sample size, why not including at least 10 subjects instead of only 8 as suggested,just because of the possibility of someone dropping out? Further, the authors might want to consider reviewing some studies that investigated the association of RFD and aging and the implications of the most rapid isometric contractions. Contractile RFD might be equally if not more sensitive to fatigue than power.
The authors put a great effort both in lab work and writing. The introduction is very extensive and somewhat hard to follow, especially the last paragraph. Methods are very detailed and followed by appropriate statistical analysis. Results and discussion detailed and extensive and similarly as introduction, sometimes hard to follow. The conclusions should provide the bottom-line of your hard work, but I don’t find it. Outline the novelty and the significance.

Thank you for your careful review of our manuscript. While inclusion of males and females, with a sufficient sample size in each group, might have been interesting, Sundberg et al. observed no sex-related differences in muscle fatigue, recovery of muscular torque or power, or voluntary activation when using a similar fatiguing protocol (Sundberg et al. 2018, JAP). Thus, we are confident that only including women in this study did not affect the generalizability of our results.

Specific comments:

Title: It seems that something like “Muscle architecture, voluntary activation, and low-frequency fatigue do not explain the greater fatigue of older compared with young women during high-velocity contractions“ would fit better to what you presented here

Thank you for this suggestion. We have changed the title of our manuscript to the one you suggested.

Line 54: A number of studies have shown that RFD rather than power has association with such risk.

Whilst some authors report an association between the rate of torque development (RTD) and fall risk with aging, other authors have reported no age-related differences in the RTD (Callahan et al. 2009, Muscle Nerve; Chung et al. 2007, JAP). One important distinction between RTD and muscular power is that RTD is often measured under isometric conditions, as we did in this study. Perhaps, then, the important component here is the rapid development of muscular force, as we highlight in Lines 58-61.

Line 61: And sarcopenia that kicks-in by the age of 40.

Sarcopenia refers to the progressive loss of muscle mass and the resulting impairment to physical function, with age (Morley et al. 2011, J Am Med Dir Assoc). Baumgartner et al. provided an operational definition of sarcopenia as being 2 standard deviations below the muscle mass of healthy young individuals (Baumgartner et al. 1998, Am J Epidemiol). Although muscle mass declines at ~1% per year after the age of ~40 years, severe loss of muscle (2 standard deviations below young healthy individuals) is only present in 5 to 13% of 60-70 year olds (Fielding et al. 2011, J Am Med Dir Assoc; von Haehling et al. 2010, J Cachexia Sarcopenia Muscle). We are not certain what the reviewer intended with this comment, but we highlight the effects of reduced muscle mass on architectural remodeling in the subsequent paragraph.

Line 184 and 209: Can you please justify positioning with the “hips at 90° and a knee angle of 100° extension (180° = full extension)”? Why not using 120 degrees in knee extension?

We chose these hip and knee angles for three reasons. Firstly, this is a comfortable sitting position for participants. Secondly, this position has been used previously in studies of age-related differences in muscle fatigue (Callahan et al. 2009, Muscle Nerve; Callahan & Kent-Braun, 2011, JAP). Lastly, we have noted that some older adults cannot reach full extension (180°) whilst sitting due to hamstring tightness. Therefore, a knee angle of 100° allowed participants to perform the contractions over a large range of motion (70°), which would not have been feasible with a more extended starting knee angle.

L230: What was the reason for sampling at 2500 Hz? Please provide a reference for data acquisition.

We had originally planned to collect EMG data throughout the fatigue protocol (although this did not work out), so we used a high sampling rate on all channels. This approach has been used previously in our lab (Callahan & Kent-Braun, 2011, JAP). Further, others have shown no difference in peak torque or the rate of torque development at sampling frequencies of 500, 1,000, and 2,000Hz (Thompson 2019, J Biomech), so we are confident the sampling frequency did not affect any measures in the current study.

L251: Why did you chose peak RTD and not “contractile RTD” as suggested by Aagaard et al 2002, 2007 etc.? RTS derived at various time intervals from the contraction onset has been shown as sensitive to both training and surgical interventions.

We reported the peak rate of torque development as a percentage of the peak torque elicited by the 80Hz tetanic contraction, per millisecond (% peak per millisecond; %pk**^.^**ms^-1^) which is consistent with previous work in our lab (Callahan et al. 2009, Muscle Nerve; Chung et al. 2007, JAP; etc). Normalizing to peak torque achieved accounts for the effects of torque production on the rate of torque development (Miller et al, 1981) and thus focuses on the speed of the contraction. Based upon Figure 2 in the paper you refer to (Aagaard et al, 2007, MSSE), our measure of peak RTD is similar to the value they reported at 50ms. Further, Aagaard et al. showed that the contractile RTD was most different between their groups at 50ms. Thus we are confident that we would have observed the same result (i.e., no difference in RTD between young and older at baseline nor with fatigue) if we had reported contractile RTD instead of peak RTD. In response to the reviewer’s comment, we now report the peak RTD throughout recovery in Table S1 (supplementary information). The group data for RTD at baseline are reported in Table 2 of the manuscript.

L283: Please provide the interpretation for Cohen’s d

We have provided qualitative interpretation of different d values on Lines 288-289, based on Cohen’s work.

Table 3: Consider presenting these data as three line-graphs.

Thank you for this suggestion. We now report these data in Figure 5.

6. PLOS authors have the option to publish the peer review history of their article ([what does this mean?](https://urldefense.proofpoint.com/v2/url?u=https-3A__journals.plos.org_plosone_s_editorial-2Dand-2Dpeer-2Dreview-2Dprocess-23loc-2Dpeer-2Dreview-2Dhistory&d=DwMGaQ&c=sJ6xIWYx-zLMB3EPkvcnVg&r=khMOZAIPugRDx2mPk882SyrBxYwUZMrH0Ns3Ow2XUos&m=UeqZnOL9tqkQWNIWl4V2FUZEUT3QfURydfRinmczEi8&s=-AfXKg3evWzJsxCczBfvUu0U-cg71T4YtuWjw18aujY&e=)). If published, this will include your full peer review and any attached files.


If you choose “no”, your identity will remain anonymous but your review may still be made public.


**Do you want your identity to be public for this peer review?** For information about this choice, including consent withdrawal, please see our [Privacy Policy](https://urldefense.proofpoint.com/v2/url?u=https-3A__www.plos.org_privacy-2Dpolicy&d=DwMGaQ&c=sJ6xIWYx-zLMB3EPkvcnVg&r=khMOZAIPugRDx2mPk882SyrBxYwUZMrH0Ns3Ow2XUos&m=UeqZnOL9tqkQWNIWl4V2FUZEUT3QfURydfRinmczEi8&s=2SyyqfuiT4_wdTN3LrEqujZTAUhWAlBihFQkYsxH9gQ&e=).

Reviewer #1: **Yes:** Patrik Drid

Reviewer #2: No

[NOTE: If reviewer comments were submitted as an attachment file, they will be attached to this email and accessible via the submission site. Please log into your account, locate the manuscript record, and check for the action link "View Attachments". If this link does not appear, there are no attachment files.]

While revising your submission, please upload your figure files to the Preflight Analysis and Conversion Engine (PACE) digital diagnostic tool, [https://pacev2.apexcovantage.com/](https://urldefense.proofpoint.com/v2/url?u=https-3A__pacev2.apexcovantage.com_&d=DwMGaQ&c=sJ6xIWYx-zLMB3EPkvcnVg&r=khMOZAIPugRDx2mPk882SyrBxYwUZMrH0Ns3Ow2XUos&m=UeqZnOL9tqkQWNIWl4V2FUZEUT3QfURydfRinmczEi8&s=5Dxzzcv0tLMsRfCVJoBBgOI_-p7RPJ_HfT5zTTSf4n0&e=). PACE helps ensure that figures meet PLOS requirements. To use PACE, you must first register as a user. Registration is free. Then, login and navigate to the UPLOAD tab, where you will find detailed instructions on how to use the tool. If you encounter any issues or have any questions when using PACE, please email PLOS at [figures@plos.org](mailto:figures@plos.org). Please note that Supporting Information files do not need this step.
